# Phospholipase C Isozymes Are Deregulated in Colorectal Cancer – Insights Gained from Gene Set Enrichment Analysis of the Transcriptome

**DOI:** 10.1371/journal.pone.0024419

**Published:** 2011-09-01

**Authors:** Stine A. Danielsen, Lina Cekaite, Trude H. Ågesen, Anita Sveen, Arild Nesbakken, Espen Thiis-Evensen, Rolf I. Skotheim, Guro E. Lind, Ragnhild A. Lothe

**Affiliations:** 1 Department of Cancer Prevention, Institute for Cancer Research, Oslo University Hospital, Oslo, Norway; 2 Centre for Cancer Biomedicine, Faculty of Medicine, University of Oslo, Oslo, Norway; 3 Department of Gastrointestinal Surgery, Oslo University Hospital, Oslo, Norway; 4 Department of Organ Transplantation, Gastroenterology, and Nephrology, Oslo University Hospital, Oslo, Norway; Naval Research Laboratory, United States of America

## Abstract

Colorectal cancer (CRC) is one of the most common cancer types in developed countries. To identify molecular networks and biological processes that are deregulated in CRC compared to normal colonic mucosa, we applied Gene Set Enrichment Analysis to two independent transcriptome datasets, including a total of 137 CRC and ten normal colonic mucosa samples. Eighty-two gene sets as described by the Kyoto Encyclopedia of Genes and Genomes database had significantly altered gene expression in both datasets. These included networks associated with cell division, DNA maintenance, and metabolism. Among signaling pathways with known changes in key genes, the “Phosphatidylinositol signaling network”, comprising part of the PI3K pathway, was found deregulated. The downregulated genes in this pathway included several members of the Phospholipase C protein family, and the reduced expression of two of these, *PLCD1* and *PLCE1*, were successfully validated in CRC biopsies (n = 70) and cell lines (n = 19) by quantitative analyses. The repression of both genes was found associated with *KRAS* mutations (*P* = 0.005 and 0.006, respectively), and we observed that microsatellite stable carcinomas with reduced *PLCD1* expression more frequently had *TP53* mutations (*P* = 0.002). Promoter methylation analyses of *PLCD1* and *PLCE1* performed in cell lines and tumor biopsies revealed that methylation of *PLCD1* can contribute to reduced expression in 40% of the microsatellite instable carcinomas. In conclusion, we have identified significantly deregulated pathways in CRC, and validated repression of *PLCD1* and *PLCE1* expression. This illustrates that the GSEA approach may guide discovery of novel biomarkers in cancer.

## Introduction

**Figure 1:**
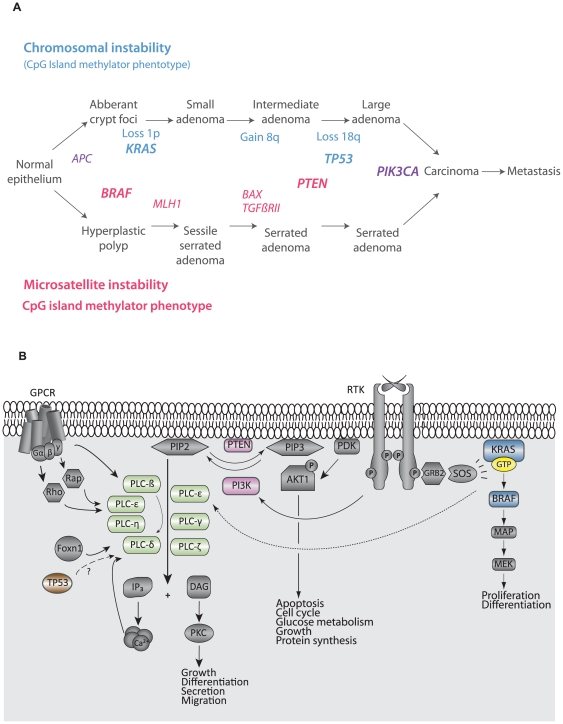
Important genes in colorectal cancer development. A) Key genes targeted in the various CRC phenotypes during development from precursor lesions to carcinomas. Genes in bold represent MAPK, PI3K, and TP53 signaling networks, and their mutation status have previously been determined [38]. B) The figure shows how part of the phosphatidyl inositol network interacts with the canonical PI3K pathway. Genes of which mutation status data are used for association analyses in the current paper are colored in blue, pink and brown. Abbreviations: GPCR, G-protein coupled receptor; RTK, receptor tyrosine kinase.

Colorectal cancer (CRC; MIM#114500) ranks third in cancer incidence world-wide with an estimated 1.2 million new cases per year [1]. According to the American Joint Committee on Cancer (AJCC) the 5-year survival rates for patients diagnosed with stage I and stage IV CRC, are 93% and 8% respectively, thus patient prognosis is highly dependent on the tumor stage at diagnosis [2]. CRC develops through distinct histopathological stages from benign precursor lesions to malignant tumors, known as the adenoma-carcinoma sequence. The development is driven by progressive accumulation of genetic and epigenetic alterations of specific genes, and conserved signaling networks that exert pleiotropic effects on the cancer cells are often targeted. Typically, malignant tumors are characterized by the molecular phenotypes microsatellite instability (MSI), chromosomal instability (CIN), and the CpG island methylator phenotype (CIMP) [3]. The most commonly altered genes during CRC development make up the basis for these phenotypes and involve several signaling pathways and networks (Figure 1A).

Messenger RNA (mRNA) expression profiling studies have provided improved understanding of the roles of several genes important in initiation and progression of cancer, including CRC [4]. Despite increasing knowledge of expression patterns of individual genes, as well as promising gene signatures, less insight has been obtained on alterations of molecular networks and signaling pathways from the large amount of data generated in such studies. By applying the statistical approach of Gene Set Enrichment Analysis (GSEA) to gene expression data, interacting genes whose expression levels are coordinately changed can be explored [5]. The GSEA method takes into consideration the expression of all genes within an annotated gene set/pathway. There are several databases and gene set definitions linking genes by their functional annotations. The most widely used is the Gene Ontology (GO) database, which is based on a combination of computational and manual assignment of genes to GO terms [6]. However, these terms do not correspond directly to known pathways. The Kyoto Encyclopedia of Genes and Genomes (KEGG) is a molecular pathway database of manually curated and frequently updated metabolic, regulatory, and signaling pathways [7]. Using this conservatively annotated database, the redundancy in the nested structure of gene associations represented by GO terms can be avoided. Compared to individual gene expression analyses, GSEA provides insight into the coordinated expression changes of genes within pathways, hence altered signaling pathways and genes herein not known to be involved in carcinogenesis could be identified.

The importance of the canonical Phosphatidylinositol 3-kinase (PI3K) pathway in cancer has been established in numerous publications during the last decade [8]. This signaling network has impact on several cancer-critical phenotypes such as cell proliferation, survival, metabolism, and tumor growth [8]. A branch of the network overlaps with the Phosphatidylinositol signaling network which is mediating signals through Phospholipase C (PLC) proteins (Figure 1B). This network probably crosstalks with the MAPK and TP53 pathways, important pathways in CRC development. The PLC protein family comprises thirteen isozymes divided into six distinct subtypes, β, γ, δ, ε, ζ, and η, based on structure and regulatory activation mechanisms. The PLC isozymes contain several common domains, as well as subtype specific domains, that can make them capable of regulating lipase-independent reactions [9]. Further, they are soluble and mainly localized in the cytosol, but become translocated to the plasma membrane in response to receptor activation [10]. At the membrane they hydrolyze phosphatidylinositol (4,5)-phosphate (PIP_2_) and generate the important second messenger diacylglycerol (DAG) and inositol 1,4,5-triphospate (IP_3_). Imbalance of phosphoinositide levels (PIP_2_ and PIP_3_) has been shown to induce defects in channel activity, trafficking, and normal cell polarity [9]. Thus, correct regulation of the phosphoinositides by e.g. PLC enzymes is essential and aberrant regulation contributes to various human disorders, including cancer [9].

By exploring gene expression of predefined biological processes and molecular circuits in two CRC and normal colonic mucosa datasets obtained using different microarray platforms, a list of pathways significantly altered by differential gene expression in CRC was identified. We selected the “Phosphatidylinositol signaling network” and two of the deregulated components therein, *PLCD1* (HGNC:9060) and *PLCE1* (HGNC:17175), for detailed studies and compared the findings with clinicopathological data and mutation status of several genes known to be altered in CRC.

## Results

### Deregulated KEGG pathways in CRCs

**Figure 2:**
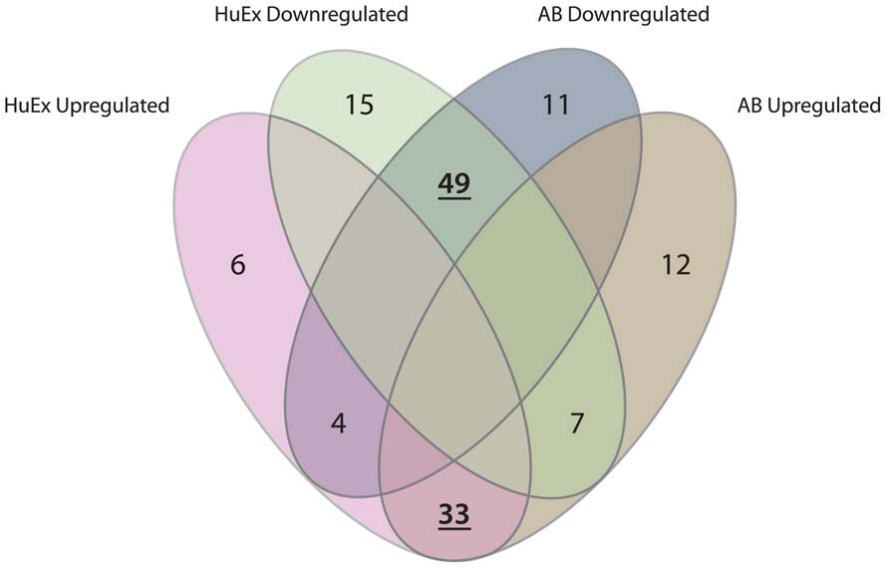
Venn diagram displaying significantly deregulated signaling pathways in the AB and HuEx gene expression datasets. The diagram shows that the results of the GSEA were highly reproducible with 33 and 49 pathways jointly up- and downregulated across the two datasets, respectively.

In total, 152 KEGG gene sets were present in both gene expression datasets. From these, GSEA revealed 33 significantly upregulated and 49 significantly downregulated biological processes and pathways in CRC compared to normal colonic mucosa (*P*≤0.05; Figure 2). Eleven significantly altered networks had opposite enrichment scores between the datasets, whereas 44 were found significantly altered in only one of the datasets. KEGG networks significantly deregulated in CRC compared to normal mucosa in both datasets are listed in Table S1 (n = 82). The fact that these pathways were found significantly altered (*P*≤0.05) in two independently analyzed datasets, obtained from different microarray platforms, gives a risk of only 1/400 for each pathway to be included as significantly altered merely by chance. Hence, no additional correction for multiple testing is performed (this also accounts for the upper part of Table 1). The 82 networks include gene sets responsible for general cancer cell traits, various metabolic circuits as well as more renowned signaling cascades such as the TP53 and MAPK pathways. Plots of gene expression levels in CRC samples *versus* normal colonic mucosa specimens for all genes in the three most upregulated and the three most downregulated networks in CRC are shown in Figure S1, and their statistically differentially expressed genes are listed in Table S2.

**Table 1:** Significantly deregulated genes within the “Phosphatidylinositol signaling system”.

Gene symbol	P-Value AB	Log fold change AB	P-Value HuEx	Log fold change HuEx
**Genes significantly altered in two datasets**				
Upregulated genes				
*** INPP5D***	5.4E-05	2.2	1.4E-03	0.8
*** PIK3R3***	2.9E-02	1.0	4.8E-03	0.9
Downregulated genes				
*** DGKA***	3.4E-02	−1.2	9.7E-03	−0.6
*** DGKD***	6.0E-04	−1.2	4.9E-06	−0.6
*** IMPA2***	1.7E-12	−1.7	1.0E-05	−1.0
*** ITPKA***	6.9E-04	−1.7	7.1E-03	−0.4
*** PIP5K1B***	1.1E-02	−14.9	1.4E-03	−1.1
*** PLCD1***	1.9E-04	−1.9	2.0E-09	−0.9
*** PLCD3***	2.1E-04	−1.3	1.4E-07	−0.8
*** PLCE1***	6.2E-05	−2.9	6.1E-05	−1.7
*** PLCG2***	1.7E-02	−1.4	1.3E-02	−0.4
**Genes significantly altered in one dataset**				
Upregulated genes				
* CDIPT*	3.0E-03	0.9	7.0E-02	0.3
* CDS2*	2.4E-02	0.7	4.5E-01	0.1
* DGKZ*	9.3E-03	0.9	6.2E-01	−0.1
* PIK3C2A*	2.4E-01	−0.4	9.4E-03	0.5
* PIK3CA*	9.7E-01	0.0	1.8E-02	0.5
* PIP5K1A*	1.0E-01	−0.9	1.7E-02	0.4
* PTPMT1*	4.8E-03	0.7	-	-
Downregulated genes				
* CALM1*	-	-	2.0E-04	−0.4
* CALM2*	5.7E-02	−0.4	1.7E-02	−0.5
* DGKB*	-	-	7.4E-04	−0.3
* IMPA1*	1.5E-02	−0.8	5.9E-02	−0.5
* INPP5A*	3.0E-01	−0.6	1.2E-04	−0.6
* INPP5J*	-	-	3.7E-04	−0.5
* INPP5K*	-	-	3.2E-02	−0.3
* ITPK1*	3.0E-01	−0.3	6.7E-04	−0.5
* PI4KA*	-	-	3.9E-02	−0.3
* PIB5PA*	4.5E-04	−1.2	-	-
* PIK3CG*	-	-	2.3E-02	−0.7
* PIK3R5*	2.7E-04	−2.0	2.3E-01	−0.1
* PIK4CA*	7.4E-04	−0.8	-	-
* PIP5K1C*	1.3E-01	0.6	1.5E-04	−0.3
* PIP5K3*	2.5E-03	−1.0	-	-
* PLCB2*	8.0E-01	0.1	1.0E-02	−0.3
* PLCD4*	-	-	1.9E-04	−0.4
* PRKCA*	1.4E-02	−0.9	7.9E-01	−0.1
* PRKCB*	5.9E-01	−0.3	1.2E-02	−0.5
* SKIP*	6.8E-03	−0.7	-	-

Genes in bold were found significantly deregulated across both gene expression datasets (upper part of the table). Due to multiple testing, *P*-values of genes significantly altered in only one of the datasets should be interpreted with caution (lower part of the table; see Results).

### The “Phosphatidylinositol signaling system”

**Figure 3:**
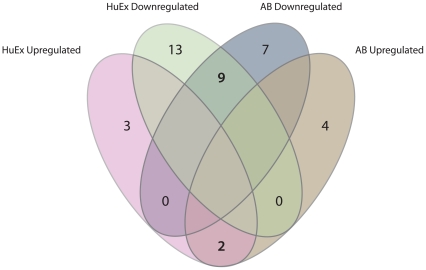
Venn diagram displaying significantly deregulated genes within the “Phosphatidylinositol signaling system”. Altogether 38 genes in the “Phosphatidylinositol signaling system” were altered in one or both dataset, eleven genes were found deregulated across both datasets.

The “Phosphatidylinositol signaling system” was one of the molecular pathways found to be downregulated in CRC compared to normal mucosa, *i.e.* having more downregulated than upregulated genes. A total of 22/59 and 27/76 genes passing the quality criteria were significantly deregulated in CRC in the AB and HuEx datasets, respectively (Figure 3, Table 1). Of the 11 genes found deregulated in both series, comprising inositol kinases, diacylglycerol kinases, and phospholipases, two were significantly upregulated, whereas nine were downregulated. Interestingly, we found four downregulated lipases (*PLCD1*, *PLCD3*, *PLCE1*, and *PLCG2)* that were closely related isozymes belonging to the Phospholipase C (PLC) family of proteins. *PLCD1* and *PLCE1*, the two PLC genes in the “Phosphatidylinositol signaling system” most significantly downregulated compared with the normal mucosa samples in the HuEx and AB datasets, respectively, were chosen for further validation.

### Validation of *PLCD1* and *PLCE1* mRNA expression

**Figure 4:**
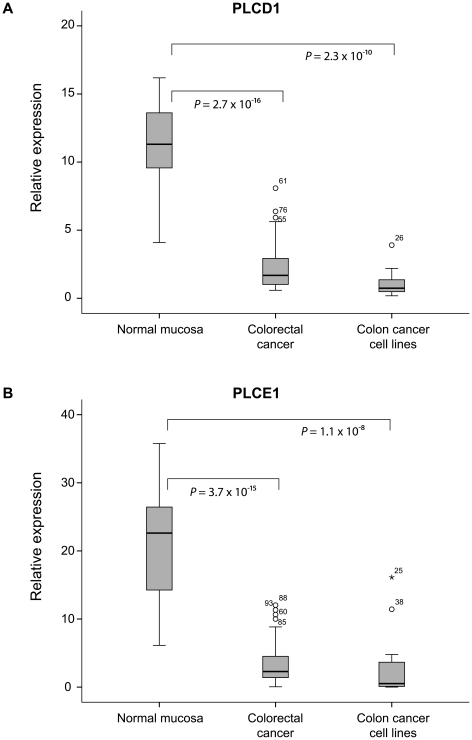
Boxplot showing markedly reduced expression levels of *PLCD1* and *PLCE1* in colorectal cancer. The expression levels of *PLCD1* and *PLCE1* in CRC and colon cancer cell lines assessed by RT-PCR were significantly downregulated compared to normal colonic mucosa.

From quantitative reverse transcription-PCR, *PLCD1* and *PLCE1* were clearly repressed in cancerous tissue samples compared with normal mucosa (*P* = 3×10^−16^ and *P* = 4×10^−15^), confirming the downregulation found in the microarray expression data (Figure 4). All the nineteen analyzed colon cancer cell lines also displayed significant repression of the two genes compared to normal mucosa (*P* = 2×10^−10^ and *P* = 1×10^−8^).

### *PLC* expression values associated to genetic and clinicopathological variables

Interestingly, there were strong associations between low expression of both *PLCD1* and *PLCE1* and *KRAS* mutations in CRC samples (Table 2). Furthermore, reduced expression levels of *PLCD1* were significantly associated with *TP53* mutations and the MSS phenotype. For both PLC genes, the expression levels were reduced in more advanced disease stages. Expression values of *PLCE1* were decreased in stage II (*P* = 0.06) and significantly decreased in stage III tumors (*P* = 0.005), compared to stage I tumors. However, this was not the case for distant metastatic disease (stage IV). The same trend was also seen for *PLCD1*, where expression levels were significantly decreased in stage III compared to stage I (*P* = 0.03). No associations were found between *PLCD1* and *PLCE1* expression and mutations in *BRAF*, *PIK3CA*, or *PTEN*. For Table 2 it should be noted that due to multiple testing (n = 16), there is a risk of false significant associations.

**Table 2:** P-values for associations of *PLCD1* and *PLCE1* mRNA expression values with clinicopathological and mutation data.

	*PLCD1*	*PLCE1*
MSI/MSS	0.01	0.47
*KRAS* mut	0.005	0.006
*BRAF* mut	0.23	0.83
*PTEN* mut	0.91	0.74
*TP53* mut	0.002	0.16
*PIK3CA* mut	0.48	0.98
Tumor stage	0.10	0.05
Localization	0.45	0.20

The expression values of *PLCD1* and *PLCE1* in CRC samples (n = 70) were compared to MSI status, mutations in various genes, tumor stage (I–IV) and localization of the tumor (right, left, rectum).

### Promoter methylation analysis of *PLCD1* and *PLCE1*

*PLCD1* and *PLCE1* could be targets for promoter methylation since their expression levels were low in CRC compared with normal colonic mucosa samples. Both genes possess CpG islands in their promoters and their expression became upregulated in cell lines treated with epigenetic reactivating agents (5-aza-2′deoxycytidine; AZA and Trichostatin A; TSA). Complete or partial methylation of the *PLCD1* promoter was found in 79% (15/19) of the colon cancer cell lines, detected by qualitative as well as quantitative methylation-specific polymerase chain reaction (MSP and qMSP, respectively). The remaining four cell lines were completely unmethylated. The methylation status of the colon cancer cell lines was confirmed by bisulfite sequencing using two non-overlapping sets of primer pairs covering altogether 69 CpG sites. There was no association between methylation and MSI-status of the cell lines. For primary carcinomas, only nine out of 70 (13%) samples were methylated, as assessed by MSP and qMSP. The majority of the tumors considered positive for methylation displayed low PMR values (median 5.69). Interestingly, eight of the nine methylated tumors were MSI, yielding a methylation frequency of 40% (8/20) in this subgroup. Furthermore, all the methylated MSI tumors had mutations in *BRAF*, whereas the methylated MSS tumor was wild type. For *PLCE1,* only one of 19 colon cancer cell lines showed promoter methylation. This was confirmed by bisulfite sequencing covering 47 CpG sites. Due to the low *PLCE1* promoter methylation frequency among the cell lines, tissue samples were not subjected to methylation analyses.

## Discussion

By exploring gene expression within predefined molecular pathways in two independent CRC transcriptome datasets, we have identified several molecular networks and components therein that are deregulated in CRC and might contribute to carcinogenesis.

The “Cell cycle” was not surprisingly the most extensively altered gene set in our analyses. Its proper regulation is essential for ensuring correct transmission of genetic information from one generation to the next, thus deregulation of several cell cycle components is defined as cancer hallmarks. Our finding is in line with several comparable studies of CRC where gene sets restricted exclusively to cancer-related pathways [11] and multiple online databases comprising more general pathways, have been explored [12]–[14]. CRCs displayed expression changes in numerous metabolic pathways, *i.e.* “Oxidative phosphorylation” was the most significantly downregulated metabolic network across both datasets, whereas metabolism of purines, pyrimidines, and N-glycans, in addition to the “Pentose phosphate pathway” were upregulated. The purine and pyrimidine metabolism were newly found to be upregulated also in adenomas compared to normal mucosa, indicating that alterations of these networks are early events in tumorigenesis [14]. Reprogramming of energy metabolism was recently launched as an emerging hallmark of cancer, and the findings are also in agreement with what Otto Warburg described half a century ago, referred to as the “Warburg effect”. He identified a shift in glucose metabolism from oxidative phosphorylation to aerobic glycolysis in tumor cells [15]. Limiting the metabolism largely to glycolysis allows conversion of glycolytic intermediates into nucleosides and amino acids. This in turn facilitates biosynthesis of macromolecules and organelles required for the increased production of new cells. Biological processes involving DNA replication and various forms of DNA repair mechanisms were also found deregulated in our datasets. These fundamental systems are frequently put out of play not only in cancer, but also in other diseases and syndromes, underscoring the severity of not being able to copy the DNA or repair mistakes in a correct manner.

In addition to more general cancer cell traits, central circuits in CRC like MAPK-, TP53-, and Phosphatidylinositol signaling systems, where usually one or a few prominent players are reported to be frequently altered at the DNA level (due to point mutations, amplifications, and/or deletions; [8], [16]), were found significantly deregulated in our analyses. This implies that altered gene expression of several less “famous” members in the pathways also contribute in colorectal carcinogenesis. In this study we observed that genes like *PIK3CA* and *PTEN* were expressed in tumor samples, but not at significantly different levels from normal colon tissue in both datasets. We and others have shown that these genes often suffer from alterations on DNA- or protein levels rather than by changes in mRNA expression [8], [17]–[19].

In order to reveal additional genes in the “Phosphatidylinositol signaling system” involved in tumorigenesis, we explored the expression level of all its components as annotated in KEGG. Both inositol phosphatases, diacylglycerol kinases, and phospholipases were found to have altered expression. Among the phospholipase enzymes, members of the Phospholipase C family were well represented, indicating an important role for this family in CRC. This was underscored by RT-PCR analyses validating that the expression levels of *PLCD1* and *PLCE1* were extensively downregulated in CRC compared to normal colonic mucosa. The repression of *PLCD1* is in accordance with the results presented by Nomoto and colleagues in 1995, where they report undetectable levels of PLCD1 protein in eight colon cancer cell lines and decreased levels in twelve of thirteen colon carcinomas compared with their paired normal mucosa sample [20]. Several groups have suggested a role for PLCD1 in regulating the cell cycle G_1_/S-checkpoint, however, there are contradicting results to whether it has an oncogenic or tumor suppressive function [21]–[23]. Based on the present findings, we suggest the latter function of *PLCD1* in CRC. Similar results have also been reported for esophageal squamous cell carcinomas (ESCC), gastric and breast cancer [21], [24]–[26]. Interestingly, the low expression levels of *PLCD1* were strongly associated with mutations in *TP53* and *KRAS* as well as to tumors of the MSS phenotype; although the biological significance of these associations remains to be determined.

The observed repression of *PLCE1*, the second gene investigated in detail, is supported by the results from Sorli *et al*. who also demonstrated significantly reduced mRNA expression levels in colon and rectum cancer samples and colon cancer cell lines, as well as by Wang and colleagues who reported a frequency of 36% loss of heterozygosity (LOH) of 10q23 where *PLCE1* is located and downregulation of PLCE1 in 21/50 colorectal cancer samples compared with matched normal tissue [27]–[29]. Furthermore, we observed that the expression levels of *PLCE1* were decreasing with more advanced stages, indicating that the repression of this gene is beneficial for cancer progression. Notably, the level appears to rise when reaching metastatic disease (stage IV), a phenomenon previously described for *PLCD1* and *PLCD3* in breast cancer [24]. Nevertheless, caution should be made when interpreting the results for the present stage IV group, as only seven tumors are included. The expression levels of *PLCD1* and *PLCE1* were correlated, and as expected, reduced expression of both genes was associated to more advanced tumor stages (data not shown). However, the individual expression levels and the expression levels of both genes combined were not correlated with patient survival (data not shown). Intriguingly, as for *PLCD1*, low expression of *PLCE1* was strongly associated with mutations in *KRAS*. Since PLCE1 is found to be both an upstream and downstream effector of Ras family proteins [30], the correlation of mutated *KRAS* with the reduced expression levels most likely plays a critical role in CRC tumorigenesis. Further, it was recently reported that overexpression of PLCE1 in a colon cancer cell line inhibited tumor cell proliferation, reduced number of colonies formed, reduced migration, and increased apoptosis [29], suggesting a tumor suppressive role for this gene in CRC.

We then investigated whether the reduced expression levels of *PLCD1* and *PLCE1* could be due to promoter hypermethylation. This is a common way to inactivate tumor suppressor genes, and both phopholipases have been put forward as candidates for promoter hypermethylation [27]. However, this has so far only been confirmed in *PLCD1* within a range of 52–62% methylation in ESCC, gastric and breast cancer [21], [25], [26]. Our results from qMSP and bisulfite sequencing cannot confirm *PLCE1* as a target for this type of epigenetic silencing, and despite high frequency of methylation in colon cancer cell lines, methylation in the *PLCD1* promoter can only explain reduced expression in a subgroup of the carcinomas. Interestingly though, we discovered that all but one of the methylated tumors were MSI, yielding a methylation frequency of nearly forty percent among carcinomas with defect mismatch repair. Additionally, the methylated MSI tumors were localized in the right side of the bowel and had *BRAF* mutation, in line with the CIMP phenotype [3].

By subjecting two CRC transcriptome datasets to GSEA, we have identified a number of statistically significant deregulated gene sets in CRC, and shown that this approach can guide discovery of novel molecular targets and biomarkers for cancer. Verification analyses of selected genes within one of the identified deregulated signaling pathways, *PLCD1* and *PLCE1*, confirmed reduced levels of these transcripts in CRC compared to normal mucosa samples. Both a tumor suppressive and tumor promoting role of these genes and protein products have been suggested in relation to cancer development, depending on tissue of origin [9]. The present data support a tumor suppressive role of *PLCD1* and *PLCE1* in CRC, which for *PLCD1* possibly can be explained by an epigenetic mechanism in microsatellite unstable carcinomas.

## Materials and Methods

### Ethics statement

Written informed consent was obtained from all subjects included. All samples were retrieved from research biobanks as a part of research projects approved according to national ethical guidelines. The biobank series have been registered according to national legislation and is approved by the Regional Committee for Medical Research Ethics (REK South-East S-09282c2009/4958, Biobank 2781; REK South: 2003, S-02126, Biobank 296-2005-174211).

### Transcriptome datasets

Two independent colorectal tissue transcriptome profiling datasets previously generated in our laboratory were used for this GSEA study [31], [32]. The raw data are accessible through the NCBI's Gene Expression Omnibus public repository for microarray data (accession numbers GSE25071 and GSE24550). The first dataset includes gene expression profiles from 46 CRCs and four normal colonic mucosa specimens obtained using the Applied Biosystems 1700 platform (Applied Biosystems by Life Technologies, Foster City, CA, USA; AB) [31]. The second gene expression dataset contains 91 CRCs and six normal colonic mucosa specimens generated using the Affymetrix GeneChip Human Exon 1.0 ST arrays (Affymetrix, Santa Clara, CA, USA; HuEx) [32]. There were no overlapping samples between the two microarray datasets. A summary of the clinical data for patients included in the datasets can be found in Table S3.

### Tissue samples and cancer cell lines

The patient samples included in target verification analyses constituted a subset of the tumors used in the HuEx dataset (n = 70), and 17 normal colonic mucosa samples (including six from the HuEx dataset). All samples were taken from patients undergoing primary surgery for CRC at Oslo University Hospital, Aker, Oslo, between 2005 and 2008. The normal colonic mucosa samples were taken from disease-free areas in the resection margin of the CRC specimens (≥10 cm from the tumor). A clinicopathological description can be found in Table S4.

Nineteen colon cancer cell lines (Co115, Colo320, EB, FRI, HCT15, HCT116, HT29, IS1, IS2, IS3, LoVo, LS1034, LS174T, RKO, SW48, SW480, TC7, TC71, and V9P) were included in the study, and are thoroughly described in [33]. These were cultured under standard conditions which will be given upon request. Six of the cell lines (HCT15, RKO, SW48, HT29, LS1034, and SW480) were subjected to epigenetic treatment using the demethylating drug AZA (Sigma-Aldrich, St. Louis, MO, USA; 1 µM for 72 hours), the histone deacetylase inhibitor TSA (Sigma-Aldrich; 0.5 µM for 12 hours), and a combination of both drugs (1 µM AZA for 72 hours and 0.5 µM TSA added the last 12 hours). In parallel, the same cell lines were cultured without treatment for 72 hours. All commercially available cell lines were authenticated using AmpFLSTR Identifiler PCR Amplification Kit (Applied Biosystems).

### Isolation of nucleic acids

DNA from fresh-frozen colonic tissue and colon cancer cell lines was extracted by a standard phenol/chloroform protocol. RNA from tumor tissue was extracted using the Qiagen AllPrep DNA/RNA Mini Kit (Qiagen GmbH, Hilden, Germany), whereas RNA from normal colonic mucosa samples and colon cancer cell lines was isolated by the Ambion RiboPure™ Kit (Life Technologies, Carlsbad, CA, USA) and Trizol (Invitrogen, Carlsbad, CA, USA), respectively. All procedures were performed according to the manufacturers' protocols.

### Gene Set Enrichment Analysis (GSEA)

For GSEA gene sets were defined according to the KEGG pathway collection (Homo sapiens (human) Release 53.0) [7]. The analysis was conducted using GSEABase package in Bioconductor/R version 2.9.2 [34]. The two microarray gene expression datasets of CRC *versus* normal colonic mucosa were independently subjected to GSEA. Data preprocessing was performed as previously described [31], [32]. Interquartile range (IQR) was used as a measure of variability. Genes with IQR <0.5 were excluded from further analyses with GSEA. For genes targeted by multiple transcript clusters (HuEx) or probes (AB), the reporter with largest variability was used. Genes not assigned to any KEGG pathways were also excluded from the analysis. A total of 3,361 and 4,797 genes with an annotated role in 205 and 220 KEGG pathways were evaluated for the AB and HuEx datasets, respectively. Pathways that included less than 10 annotated genes were removed from further analysis, leaving 167 and 185 pathways in the respective datasets. Two-sided, two-class t-tests assuming equal variances across CRC and normal samples were performed for all included genes. A gene set enrichment score was calculated as the sum of observed t-statistics for the genes in the set, divided by the square root of the number of genes in the set. Gene set *P*-values were computed based on the vector of probabilities of the t-statistics across the included genes [35].

### Validation of PLCD1 and PLCE1 expression levels by quantitative Reverse Transcription-PCR

Total RNA from CRC tissue and normal mucosa samples was converted to cDNA using the High-Capacity cDNA Reverse Transcription Kit (Applied Biosystems). The cDNA of two endogenous controls, *ACTB* (Hs99999903_m1) and *GUSB* (Hs99999908_m1), as well as cDNA of the genes *PLCD1* (Hs00979908_m1) and *PLCE1* (Hs00275279_m1) was amplified separately in 384 well-plates as described by the manufacturer (Applied Biosystems). Gene expression was measured real-time using the 7900 HT Sequence Detection System (Applied Biosystems). The samples were analyzed in triplicates and the median value was used for data analysis. A serial dilution of cDNA from the Universal Human Reference RNA (Agilent, Santa Clara, CA, USA) was used to generate the standard curve. The expression levels of *PLCD1* and *PLCE1* were normalized against the mean value of the endogenous controls.

### Bisulfite treatment and promoter methylation assay design

Prior to qualitative and quantitative MSP and bisulfite sequencing, 1.3 µg DNA from each sample was sodium bisulfite treated using the Epitect Bisulfite Kit (Qiagen) according to the manufacturer's protocol. The DNA cleansing was performed using a QiaCube (Qiagen), and the final elution volume was set to 40 µl.

Primers for MSP and bisulfite sequencing were designed using the Methyl Primer Express v.1 software (Applied Biosystems), whereas primers and probe for the qMSPs were designed using the Primer Express v3.0 software (Applied Biosystems). Primers were purchased from Medprobe (Oslo, Norway) and the probe from Applied Biosystems. Primer and probe sequences, product fragment lengths and conditions are listed in Table S5. See Figure S2 for primer and probe locations.

### Qualitative methylation-specific polymerase chain reaction

The promoter methylation status of both *PLCD1* (NM_006225.3) and *PLCE1* (NM_016341) was analyzed by MSP using the HotStarTaq DNA Polymerase (Qiagen). Positive results were verified by a second round of PCR. Bisulfite modified SssI methyltransferase (New England Biolabs, Ipswich, MA, USA) treated human placental DNA (Sigma-Aldrich, St.Louis, MO, USA) and DNA from normal lymphocytes served as positive controls for the methylated and unmethylated reaction, respectively. Water was used as negative control in both reactions.

### Quantitative methylation-specific polymerase chain reaction

The *PLCD1* promoter was also analyzed by quantitative MSP according to a protocol previously described [36]. *ALUC4* was used as an internal reference to normalize for the amount of input DNA. The level of DNA methylation was calculated as Percent of Methylated Reference (PMR) by the following equation: [(*PLCD1*/*ALU*)^sample^ / (*PLCD1*/*ALU*)^positive control^] ×100. Samples with a PMR value exceeding the highest values of normal mucosa samples, i.e. PMR>0, were considered methylated.

### Bisulfite sequencing

Bisulfite sequencing of the *PLCD1* and *PLCE1* gene promoters were performed in nineteen colon cancer cell lines. Seventy CpG sites in the *PLCD1* promoter were analyzed, including the MSP and qMSP primer and probe binding sites (see Figure S2). For the *PLCE1* promoter 47 CpG sites were analyzed, including the MSP primer binding sites. The bisulfite sequencing was carried out as described in Lind *et al*. [37].

### Molecular phenotypes and gene mutation status

Microsatellite instability/stability and mutation status of all exons of *TP53* (HGNC:11998) and *PTEN* (HGNC:9588), as well as of mutation hot spots in *KRAS* (HGNC:6407; codon 12, 13, and 61), *BRAF* (HGNC:1097; codon 600), and *PIK3CA* (HGNC:8975; exon 9 and 20) have previously been determined (Table S4) [38].

### Statistics on clinicopathological data

Statistical analyses related to clinicopathological and mutational data were conducted using PASW Statistics v.18.0 (SPSS Inc., IL, USA). Associations between gene expression levels and clinicopathological features were analyzed by Mann-Whitney-Wilcoxon (MWW) test or Kruskal-Wallis test. The MWW test was also used to examine expression level differences between normal colonic mucosa, CRC samples, and colon cancer cell lines. All reported *P*-values are two-sided, and values ≤0.05 were considered statistically significant.

## Supporting Information

Figure S1 **Plots showing gene expression of the most significantly up- and downregulated pathways.** Gene expression in CRC versus normal colonic mucosa for the three most significantly up- and downregulated pathways in two gene expression datasets, A–C and D–F, respectively. The plots are based on values from the HuEx dataset, but are representative also for the AB dataset.
(TIF)

Figure S2 **Primers and probe location relative to transcription start site.** Promoter region of A) *PLCD1* (NM_006225.3) and B) *PLCE1* (NM_016341) with location of all primers and the probe used in the present study. Vertical bars represent CpG sites, whereas +1 designate transcription start site. Abbreviations: Bisulf.seq, bisulfite sequencing; MSP, qualitative methylation-specific polymerase chain reaction; qMSP, quantitative methylation-specific polymerase chain reaction; ps, present study; s, sense; as, antisense. ^a^Primers from Hu *et.al*, Oncogene, 28, 2009.
(TIF)

Table S1 **Significantly deregulated KEGG pathways in CRC compared to normal colonic mucosa across two gene expression datasets.** ^*^the total number of genes included in each pathway after quality controls ^§^ values less than 2.6E-162.
(DOC)

Table S2 **Genes significantly deregulated in the most significantly up- and downregulated pathways in CRC.** The three most significantly upregulated pathways with the significantly differentially expressed genes in each/both datasets are presented in the upper part of the table. Similarly, the lower part of the table lists the three most significantly downregulated pathways and the differentially expressed genes. Of notice, KEGG pathway 00980: Metabolism of xenobiotics by cytochrome P450 were more downregulated than KEGG pathways 00830 and 00280 displayed in the table, but were due to substantial overlap with KEGG pathway 00982: Drug metabolism – cytochrome P450 replaced with the next pathways on the list.
(DOC)

Table S3 **Summary of patient clinical data in the transcriptome datasets.** ^a^Tumor stage according to Union for International Cancer Control (UICC)/American Joint Committee on Cancer (AJCC) staging system.
(DOC)

Table S4 **Clinical, pathological, and molecular data for patient samples included in the validation and methylation analyses.** ^a^Tumor stage according to UICC/AJCC staging system Abbreviations: M, male; F, female; MMR, mismatch repair; MSI, microsatellite instable; MSS, microsatellite stable; Wt, wild type; Mut, mutated; Unmeth, unmethylated; Meth, methylated.
(DOC)

Table S5 **Primers and probe used for qualitative and quantitative methylation-specific polymerase chain reaction and bisulfite sequencing.** For *PLCD1* (NM_006225.3) the MSP primers were obtained from Hu *et al.* (Oncogene, 28, 2009, p.2466-75) whereas the bisulfite sequencing was performed with primers from Hu *et al.* combined with primers specifically designed for the present study. For *PLCE1* (NM_016341), all primers were designed for the present study. Abbreviations: MSP, methylation-specific polymerase chain reaction; M, methylated-specific primers; U, unmethylated-specific primers; qMSP, quantitative methylation-specific polymerase chain reaction; MGB, minor groove binder; BS, bisulfite sequencing.
(DOC)
