# Supplementary figures and images for: Phospholipase C Isozymes Are Deregulated in Colorectal Cancer – Insights Gained from Gene Set Enrichment Analysis of the Transcriptome
Source: PLoS One. 2011 Sep 1;6(9):e24419. doi: 10.1371/journal.pone.0024419 (PMC3164721; doi:10.1371/journal.pone.0024419)

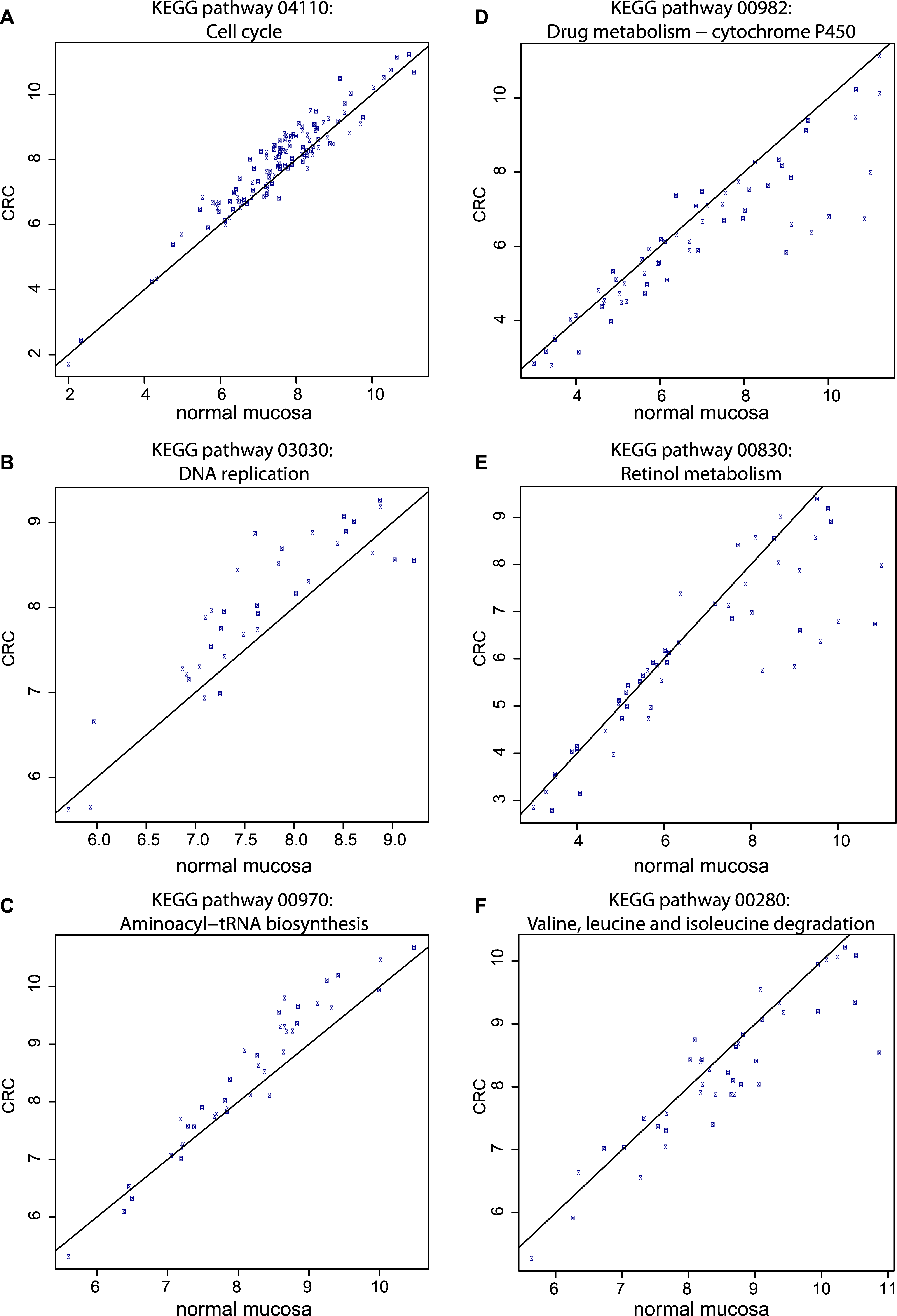

Supplement: Figure S1 — Plots showing gene expression of the most significantly up- and downregulated pathways. Gene expression in CRC versus normal colonic mucosa for the three most significantly up- and downregulated pathways in two gene expression datasets, A–C and D–F, respectively. The plots are based on values from the HuEx dataset, but are representative also for the AB dataset. (TIF) [file pone.0024419.s001.tif]

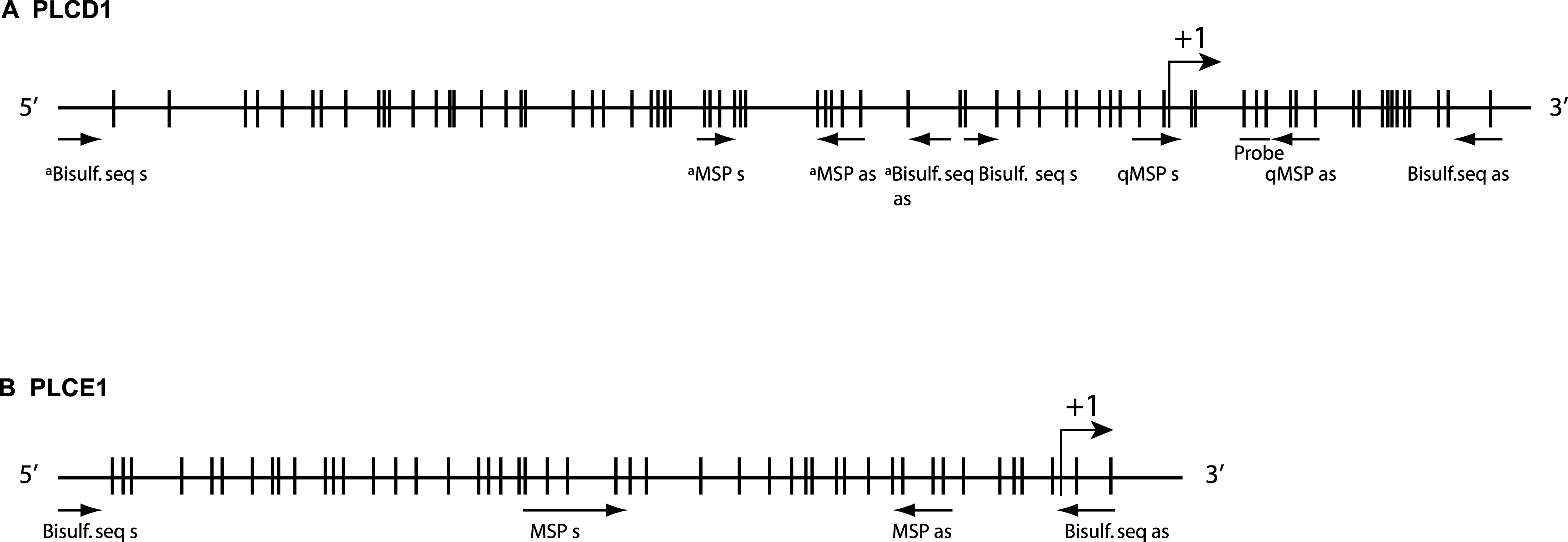

Supplement: Figure S2 — Primers and probe location relative to transcription start site. Promoter region of A) PLCD1 (NM_006225.3) and B) PLCE1 (NM_016341) with location of all primers and the probe used in the present study. Vertical bars represent CpG sites, whereas +1 designate transcription start site. Abbreviations: Bisulf.seq, bisulfite sequencing; MSP, qualitative methylation-specific polymerase chain reaction; qMSP, quantitative methylation-specific polymerase chain reaction; ps, present study; s, sense; as, antisense. aPrimers from Hu et.al, Oncogene, 28, 2009. (TIF) [file pone.0024419.s002.tif]
