# Supplementary material for: Phospholipase C Isozymes Are Deregulated in Colorectal Cancer – Insights Gained from Gene Set Enrichment Analysis of the Transcriptome
Source: PLoS One. 2011 Sep 1;6(9):e24419. doi: 10.1371/journal.pone.0024419 (PMC3164721; doi:10.1371/journal.pone.0024419)
Supplement: Table S1 — Significantly deregulated KEGG pathways in CRC compared to normal colonic mucosa across two gene expression datasets. *the total number of genes included in each pathway after quality controls § values less than 2.6E-162. (DOC) [file pone.0024419.s003.doc]

Table S1: Significantly deregulated KEGG pathways in CRC compared to normal colonic mucosa across two gene expression datasets

| **KEGG pathway number** | | **KEGG pathway name** | **AB z-score** | **AB *P*-values** | **Number of genes^*^** | | **HuEx z-score** | | **HuEx *P*-values** | | **Number of genes^*^** | |  |
| --- | --- | --- | --- | --- | --- | --- | --- | --- | --- | --- | --- | --- | --- |
| **KEGG Pathways upregulated in CRC** | | | | | | | | | | | | | |
| 05330 | Allograft rejection | | 6.5 | 2.9E-11 | | 26 | | 1.9 | | 2.9E-02 | | 32 | |
| 00970 | Aminoacyl-tRNA biosynthesis | | 8.7 | 0.0^§^ | | 38 | | 10.5 | | 0.0^§^ | | 40 | |
| 04612 | Antigen processing and presentation | | 6.9 | 3.2E-12 | | 58 | | 4.6 | | 2.5E-06 | | 75 | |
| 05310 | Asthma | | 7.2 | 2.1E-13 | | 19 | | 2.4 | | 8.2E-03 | | 26 | |
| 03022 | Basal transcription factors | | 3.3 | 5.4E-04 | | 28 | | 2.4 | | 7.3E-03 | | 32 | |
| 03410 | Base excision repair | | 6.3 | 1.8E-10 | | 33 | | 5.1 | | 1.9E-07 | | 34 | |
| 04514 | Cell adhesion molecules (CAMs) | | 2.1 | 2.0E-02 | | 95 | | 2.9 | | 2.0E-03 | | 126 | |
| 04110 | Cell cycle | | 16.7 | 0.0^§^ | | 102 | | 14.2 | | 0.0^§^ | | 123 | |
| 00532 | Chondroitin sulfate biosynthesis | | 9.7 | 0.0^§^ | | 18 | | 2.8 | | 2.7E-03 | | 22 | |
| 04610 | Complement and coagulation cascades | | 5.7 | 5.5E-09 | | 46 | | 2.9 | | 2.0E-03 | | 68 | |
| 03030 | DNA replication | | 11.9 | 0.0^§^ | | 34 | | 8.6 | | 0.0^§^ | | 35 | |
| 04512 | ECM-receptor interaction | | 3.7 | 1.0E-04 | | 63 | | 11.1 | | 0.0^§^ | | 79 | |
| 05332 | Graft-versus-host disease | | 6.0 | 9.5E-10 | | 29 | | 2.1 | | 1.8E-02 | | 34 | |
| 00534 | Heparan sulfate biosynthesis | | 2.8 | 2.6E-03 | | 12 | | 3.4 | | 3.0E-04 | | 26 | |
| 03440 | Homologous recombination | | 3.9 | 4.7E-05 | | 25 | | 4.9 | | 6.0E-07 | | 28 | |
| 00310 | Lysine degradation | | 2.8 | 2.9E-03 | | 43 | | 2.8 | | 2.3E-03 | | 44 | |
| 03430 | Mismatch repair | | 6.6 | 1.5E-11 | | 20 | | 6.1 | | 6.8E-10 | | 23 | |
| 00510 | N-Glycan biosynthesis | | 4.8 | 7.3E-07 | | 39 | | 5.4 | | 3.2E-08 | | 43 | |
| 03450 | Non-homologous end-joining | | 5.2 | 1.1E-07 | | 13 | | 5.2 | | 8.1E-08 | | 13 | |
| 03420 | Nucleotide excision repair | | 10.5 | 0.0^§^ | | 38 | | 5.6 | | 9.3E-09 | | 44 | |
| 00670 | One carbon pool by folate | | 6.9 | 2.8E-12 | | 16 | | 5.9 | | 1.4E-09 | | 16 | |
| 04115 | p53 signaling pathway | | 5.0 | 3.7E-07 | | 60 | | 5.8 | | 2.5E-09 | | 68 | |
| 05130 | Pathogenic Escherichia coli infection - EHEC | | 3.9 | 5.5E-05 | | 43 | | 2.2 | | 1.5E-02 | | 50 | |
| 00030 | Pentose phosphate pathway | | 9.3 | 0.0^§^ | | 23 | | 5.4 | | 4.2E-08 | | 26 | |
| 03050 | Proteasome | | 22.1 | 0.0^§^ | | 40 | | 2.3 | | 1.2E-02 | | 43 | |
| 00230 | Purine metabolism | | 8.2 | 1.1E-16 | | 121 | | 1.7 | | 4.8E-02 | | 152 | |
| 00240 | Pyrimidine metabolism | | 12.0 | 0.0^§^ | | 83 | | 3.1 | | 9.7E-04 | | 95 | |
| 03020 | RNA polymerase | | 7.7 | 7.0E-15 | | 23 | | 4.3 | | 9.8E-06 | | 29 | |
| 05222 | Small cell lung cancer | | 4.0 | 3.7E-05 | | 73 | | 6.6 | | 2.5E-11 | | 85 | |
| 00100 | Steroid biosynthesis | | 6.2 | 2.5E-10 | | 23 | | 8.4 | | 0.0^§^ | | 17 | |
| 05322 | Systemic lupus erythematosus | | 11.6 | 0.0^§^ | | 85 | | 5.5 | | 2.0E-08 | | 121 | |
| 04940 | Type I diabetes mellitus | | 5.8 | 4.1E-09 | | 33 | | 4.0 | | 3.7E-05 | | 38 | |
| 04120 | Ubiquitin mediated proteolysis | | 11.5 | 0.0^§^ | | 114 | | 5.7 | | 6.0E-09 | | 129 | |
| **KEGG Pathways downregulated in CRC** | | | | | | | | | | | |  | |
| 00641 | 3-Chloroacrylic acid degradation | | -5.9 | 2.0E-09 | | 12 | | -3.7 | | 1.1E-04 | | 12 | |
| 02010 | ABC transporters | | -7.1 | 5.1E-13 | | 33 | | -7.5 | | 2.9E-14 | | 44 | |
| 05221 | Acute myeloid leukemia | | -2.9 | 1.9E-03 | | 48 | | -5.5 | | 2.2E-08 | | 57 | |
| 04920 | Adipocytokine signaling pathway | | -1.8 | 3.2E-02 | | 55 | | -6.0 | | 1.2E-09 | | 66 | |
| 05010 | Alzheimer's disease | | -2.7 | 3.9E-03 | | 133 | | -20.4 | | 5.8E-93 | | 156 | |
| 00150 | Androgen and estrogen metabolism | | -2.7 | 3.6E-03 | | 30 | | -5.1 | | 2.1E-07 | | 38 | |
| 04210 | Apoptosis | | -2.8 | 2.2E-03 | | 73 | | -2.4 | | 8.0E-03 | | 86 | |
| 00590 | Arachidonic acid metabolism | | -1.8 | 3.3E-02 | | 46 | | -3.4 | | 2.8E-04 | | 55 | |
| 04360 | Axon guidance | | -5.5 | 2.4E-08 | | 96 | | -5.2 | | 7.6E-08 | | 124 | |
| 00650 | Butanoate metabolism | | -7.0 | 1.5E-12 | | 35 | | -5.9 | | 2.4E-09 | | 31 | |
| 04020 | Calcium signaling pathway | | -9.8 | 7.6E-23 | | 110 | | -8.0 | | 5.4E-16 | | 175 | |
| 04710 | Circadian rhythm - mammal | | -4.1 | 2.0E-05 | | 12 | | -3.6 | | 1.8E-04 | | 13 | |
| 00020 | Citrate cycle (TCA cycle) | | -5.1 | 2.0E-07 | | 29 | | -7.5 | | 4.4E-14 | | 30 | |
| 00982 | Drug metabolism - cytochrome P450 | | -10.7 | 8.2E-27 | | 46 | | -13.7 | | 7.0E-43 | | 62 | |
| 04012 | ErbB signaling pathway | | -5.0 | 3.2E-07 | | 67 | | -5.8 | | 2.7E-09 | | 85 | |
| 00071 | Fatty acid metabolism | | -7.8 | 2.3E-15 | | 39 | | -9.1 | | 4.2E-20 | | 40 | |
| 00361 | gamma-Hexachlorocyclohexane degradation | | -3.5 | 2.1E-04 | | 12 | | -1.7 | | 4.9E-02 | | 18 | |
| 04540 | Gap junction | | -3.1 | 8.6E-04 | | 71 | | -5.2 | | 8.5E-08 | | 89 | |
| 05214 | Glioma | | -3.7 | 1.1E-04 | | 51 | | -3.5 | | 2.3E-04 | | 63 | |
| 04912 | GnRH signaling pathway | | -8.7 | 1.6E-18 | | 81 | | -7.6 | | 1.2E-14 | | 99 | |
| 00562 | Inositol phosphate metabolism | | -8.1 | 2.0E-16 | | 38 | | -6.9 | | 3.1E-12 | | 54 | |
| 04910 | Insulin signaling pathway | | -4.3 | 7.8E-06 | | 104 | | -8.7 | | 2.2E-18 | | 134 | |
| 00591 | Linoleic acid metabolism | | -4.2 | 1.2E-05 | | 21 | | -1.9 | | 3.1E-02 | | 27 | |
| 04730 | Long-term depression | | -4.7 | 1.1E-06 | | 54 | | -3.7 | | 1.2E-04 | | 70 | |
| 04720 | Long-term potentiation | | -8.1 | 3.9E-16 | | 53 | | -7.1 | | 6.5E-13 | | 71 | |
| 04010 | MAPK signaling pathway | | -7.8 | 4.2E-15 | | 203 | | -5.9 | | 1.6E-09 | | 263 | |
| 04916 | Melanogenesis | | -4.0 | 3.0E-05 | | 77 | | -4.0 | | 3.1E-05 | | 102 | |
| 05218 | Melanoma | | -6.6 | 1.5E-11 | | 52 | | -2.1 | | 1.7E-02 | | 69 | |
| 00980 | Metabolism of xenobiotics by   cytochrome P450 | | -9.8 | 4.3E-23 | | 47 | | -14.1 | | 2.5E-45 | | 62 | |
| 00760 | Nicotinate and nicotinamide metabolism | | -2.2 | 1.3E-02 | | 20 | | -4.7 | | 1.3E-06 | | 24 | |
| 00910 | Nitrogen metabolism | | -6.0 | 9.7E-10 | | 20 | | -5.8 | | 2.7E-09 | | 24 | |
| 05223 | Non-small cell lung cancer | | -1.8 | 3.4E-02 | | 46 | | -3.8 | | 7.3E-05 | | 54 | |
| 00190 | Oxidative phosphorylation | | -2.9 | 1.6E-03 | | 103 | | -27.1 | | 2.6E-162 | | 113 | |
| 05012 | Parkinson's disease | | -4.0 | 2.6E-05 | | 100 | | -26.6 | | 9.0E-156 | | 111 | |
| 00040 | Pentose and glucuronate interconversions | | -7.9 | 1.3E-15 | | 15 | | -9.9 | | 2.9E-23 | | 17 | |
| 04070 | Phosphatidylinositol signaling system | | -5.8 | 2.5E-09 | | 59 | | -7.0 | | 1.8E-12 | | 76 | |
| 00860 | Porphyrin and chlorophyll metabolism | | -3.5 | 2.2E-04 | | 28 | | -7.2 | | 3.0E-13 | | 33 | |
| 03320 | PPAR signaling pathway | | -1.8 | 3.4E-02 | | 53 | | -7.1 | | 6.1E-13 | | 67 | |
| 00640 | Propanoate metabolism | | -4.9 | 3.9E-07 | | 30 | | -5.9 | | 1.6E-09 | | 32 | |
| 04810 | Regulation of actin cytoskeleton | | -4.1 | 1.9E-05 | | 161 | | -3.5 | | 2.0E-04 | | 208 | |
| 04140 | Regulation of autophagy | | -3.5 | 2.4E-04 | | 21 | | -6.0 | | 9.7E-10 | | 32 | |
| 00830 | Retinol metabolism | | -10.3 | 2.7E-25 | | 32 | | -8.6 | | 2.6E-18 | | 54 | |
| 00500 | Starch and sucrose metabolism | | -8.4 | 1.8E-17 | | 37 | | -10.4 | | 1.2E-25 | | 41 | |
| 04660 | T cell receptor signaling pathway | | -3.3 | 5.4E-04 | | 66 | | -3.8 | | 8.6E-05 | | 106 | |
| 04742 | Taste transduction | | -11.3 | 6.8E-30 | | 24 | | -6.2 | | 3.5E-10 | | 50 | |
| 04530 | Tight junction | | -5.2 | 1.1E-07 | | 103 | | -2.4 | | 8.9E-03 | | 129 | |
| 04930 | Type II diabetes mellitus | | -7.7 | 5.7E-15 | | 32 | | -2.3 | | 9.5E-03 | | 47 | |
| 00280 | Valine, leucine and isoleucine degradation | | -9.9 | 2.3E-23 | | 45 | | -9.5 | | 1.4E-21 | | 42 | |
| 04370 | VEGF signaling pathway | | -2.6 | 5.1E-03 | | 62 | | -3.1 | | 9.8E-04 | | 72 | |
