# Supplementary material for: Phospholipase C Isozymes Are Deregulated in Colorectal Cancer – Insights Gained from Gene Set Enrichment Analysis of the Transcriptome
Source: PLoS One. 2011 Sep 1;6(9):e24419. doi: 10.1371/journal.pone.0024419 (PMC3164721; doi:10.1371/journal.pone.0024419)
Supplement: Table S2 — Genes significantly deregulated in the most significantly up- and downregulated pathways in CRC. The three most significantly upregulated pathways with the significantly differentially expressed genes in each/both datasets are presented in the upper part of the table. Similarly, the lower part of the table lists the three most significantly downregulated pathways and the differentially expressed genes. Of notice, KEGG pathway 00980: Metabolism of xenobiotics by cytochrome P450 were more downregulated than KEGG pathways 00830 and 00280 displayed in the table, but were due to substantial overlap with KEGG pathway 00982: Drug metabolism – cytochrome P450 replaced with the next pathways on the list. (DOC) [file pone.0024419.s004.doc]

**Table S2: Genes significantly deregulated in the most significantly up- and downregulated pathways in CRC**

| **KEGG pathway number and name** |  | **AB** | **AB/HuEx** | **HuEx** |
| --- | --- | --- | --- | --- |
| KEGG Pathways upregulated in colorectal cancer | | |  |  |
| 04110 Cell cycle | Upregulated genes | *ANAPC10, ANAPC5, ANAPC7, CCND1, CDC14B, CDC2, CDC45L, CDK4, CDKN2D, GSK3B, MAD2L1, ORC6L, PCNA, PTTG2, SMAD2, SMC1L1, TGFB1* | *BUB1, CCNA2, CCNB1, CCNB2, CDC20, CDC25B, CDC6, CHEK1, CUL1, DBF4, ESPL1, MAD2L2, MCM2, MCM3, MCM4, MCM5, MCM6, ORC3L, ORC5L, PLK1, PTTG1, RB1, SKP2, TFDP1, YWHAG, YWHAQ* | *ANAPC1, ATR, BUB1B, BUB3, CDC23, CDC27, CDC7, CDK2, CDK6, HDAC2, MCM7, MYC , ORC1L, PKMYT1, PRKDC,RAD21, RBL1, SMC1A, SMC3, STAG2, TFDP2, TGFB2, TTK* |
|  | Downregulated genes |  | *CDC14A* | *ABL1, CCND3, CDKN1A, CDKN2B, E2F2, SKP1, YWHAE* |
| 03030 DNA replication | Upregulated genes | *FLJ11712, PCNA, PRIM1, PRIM2A, RFC2, RNASEH1, RNASEH2A , RPA3, SSBP1* | *FEN1, LIG1, MCM2, MCM3, MCM4, MCM5,MCM6, POLA2, POLD2, POLE3, RFC3* | *MCM7, POLD1, PRIM2, RFC1* |
|  | Downregulated genes | *POLD3* | *POLD4, RPA4* |  |
| 00970 Aminoacyl-tRNA biosynthesis | Upregulated genes | *CARS, FARS2, FLJ12118 , KIAA1970, MTFMT, SARS2, YARS2* | *AARS, DARS, GARS, IARS, KARS, WARS, YARS* | *DARS2, EPRS, FARSA, FARSB, LARS, MARS, RARS, SARS, TARS, TARS2* |
|  | Downregulated genes | *NARS, PARSL* |  | *PSTK, RARS2, TARSL2* |
| KEGG Pathways downregulated in colorectal cancer | | |  |  |
| 00982 Drug metabolism - cytochrome P450 | Upregulated genes | *GSTO1, MGST2* |  | *ALDH1A3, FMO3* |
|  | Downregulated genes | *CYP2C18, CYP2C8, CYP2D6, FMO4, UGT1A10, UGT1A8, UGT2A1, UGT2B4* | *ADH1A, ADH1B, ADH1C, CYP2C9, GSTA1, GSTA2, GSTA3, GSTM5, MAOA, MGST3, UGT2A3* | *AOX1 , CYP3A4, CYP3A5, FMO5, GSTK1, GSTM1, GSTM2, GSTM3, GSTM4, GSTZ1, MAOB, MGST1, UGT1A9, UGT2B10, UGT2B11, UGT2B15, UGT2B17, UGT2B7* |
| 00830 Retinol metabolism | Upregulated genes | *RDH11* |  | *DGAT2* |
|  | Downregulated genes | *CYP26B1, CYP2C18, CYP2C8, RETSAT, UGT1A10, UGT1A8, UGT2A1, UGT2B4* | *ADH1A, ADH1B, ADH1C, CYP2C9, DHRS9, RDH5, UGT2A3* | *CYP3A4, CYP3A5, DGAT1, DHRS4, RETSAT, UGT1A9, UGT2B10, UGT2B11, UGT2B15, UGT2B17, UGT2B7* |
| 00280 Valine, leucine and isoleucine degradation | Upregulated genes | *HADH2* |  | *ALDH7A1* |
|  | Downregulated genes | *ALDH3A2, BCKDHA, DLD, EHHADH, IVD, OXCT2* | *ACAA1, ACAA2, ACADM, ACADS, ACADSB, ACAT1, HMGCL, HMGCS2* | *ACAD8, AOX1, AUH, BCAT2, ECHS1, HADH, HIBCH, MCEE, PCCA* |
