# Supplementary material for: Phospholipase C Isozymes Are Deregulated in Colorectal Cancer – Insights Gained from Gene Set Enrichment Analysis of the Transcriptome
Source: PLoS One. 2011 Sep 1;6(9):e24419. doi: 10.1371/journal.pone.0024419 (PMC3164721; doi:10.1371/journal.pone.0024419)
Supplement: Table S3 — Summary of patient clinical data in the transcriptome datasets. aTumor stage according to Union for International Cancer Control (UICC)/American Joint Committee on Cancer (AJCC) staging system. (DOC) [file pone.0024419.s005.doc]

**Table S3**: Summary of patient clinical data in the transcriptome datasets

|  | **Dataset** | |
| --- | --- | --- |
|  | **AB** | **HuEx** |
| **Tissue type** |  |  |
| CRC | 46 | 91 |
| Normal | 4 | 6 |
| **Sex** |  |  |
| Male | 25 | 43 |
| Female | 21 | 48 |
| **Age** |  |  |
| Average age at diagnosis | 58 | 68 |
| Range | 28-87 | 34-93 |
| **Tumor stage^a^** |  |  |
| I | 9 | 24 |
| II | 13 | 31 |
| III | 18 | 26 |
| IV | 6 | 10 |
| **Localization** |  |  |
| Proximal | 14 | 43 |
| Distal | 17 | 21 |
| Rectum | 15 | 27 |
