# Supplementary material for: Phospholipase C Isozymes Are Deregulated in Colorectal Cancer – Insights Gained from Gene Set Enrichment Analysis of the Transcriptome
Source: PLoS One. 2011 Sep 1;6(9):e24419. doi: 10.1371/journal.pone.0024419 (PMC3164721; doi:10.1371/journal.pone.0024419)
Supplement: Table S4 — Clinical, pathological, and molecular data for patient samples included in the validation and methylation analyses. aTumor stage according to UICC/AJCC staging system Abbreviations: M, male; F, female; MMR, mismatch repair; MSI, microsatellite instable; MSS, microsatellite stable; Wt, wild type; Mut, mutated; Unmeth, unmethylated; Meth, methylated. (DOC) [file pone.0024419.s006.doc]

**Table S4: Clinical, pathological, and molecular data for patient samples included in the validation and methylation analyse**s

| **Patient ID** | **Sex** | **Age at diagnosis** | **Tumor stage^a^** | **Tumor location** | **MMR deficiency** | ***PLCD1* methylation status** | **Mutation status** | | | | |
| --- | --- | --- | --- | --- | --- | --- | --- | --- | --- | --- | --- |
|  |  |  |  |  |  |  | ***KRAS*** | ***BRAF*** | ***PTEN*** | ***TP53*** | ***PIK3CA*** |
| 015 | F | 66 | IV | Proximal | MSI | Meth | Wt | Mut | Wt | Wt | Wt |
| 030 | F | 84 | II | Proximal | MSI | Meth | Wt | Mut | Wt | Wt | Wt |
| 031 | F | 67 | II | Distal | MSI | Meth | Wt | Mut | Wt | Wt | Wt |
| 045 | M | 75 | I | Proximal | MSI | Meth | Wt | Mut | Wt | Wt | Wt |
| 115 | F | 57 | I | Proximal | MSI | Meth | Wt | Mut | Mut | Wt | Mut |
| 118 | F | 81 | II | Proximal | MSI | Meth | Wt | Mut | Wt | Wt | Wt |
| 144 | F | 67 | III | Proximal | MSI | Meth | Wt | Mut | Wt | Wt | Wt |
| 147 | F | 63 | II | Proximal | MSI | Meth | Wt | Mut | Wt | Wt | Wt |
| 007 | F | 87 | I | Rectum | MSI | Unmeth | Mut | Wt | Wt | Mut | Wt |
| 010 | M | 63 | II | Proximal | MSI | Unmeth | Mut | Wt | Wt | Wt | Mut |
| 012 | F | 84 | II | Proximal | MSI | Unmeth | Wt | Mut | Mut | Wt | Wt |
| 032 | M | 69 | I | Proximal | MSI | Unmeth | Wt | Mut | Wt | Wt | Wt |
| 066 | F | 84 | II | Proximal | MSI | Unmeth | Wt | Mut | Mut | Mut | Mut |
| 072 | M | 64 | III | Proximal | MSI | Unmeth | Mut | Wt | Wt | Wt | Wt |
| 080 | F | 89 | I | Proximal | MSI | Unmeth | Wt | Mut | Wt | Wt | Wt |
| 085 | F | 68 | I | Distal | MSI | Unmeth | Wt | Mut | Wt | Wt | Wt |
| 086 | F | 70 | II | Proximal | MSI | Unmeth | Mut | Wt | Wt | Wt | Wt |
| 088 | M | 78 | II | Rectum | MSI | Unmeth | Wt | Wt | Wt | Mut | Wt |
| 117 | F | 74 | II | Proximal | MSI | Unmeth | Wt | Mut | Mut | Wt | Wt |
| 127 | F | 92 | II | Proximal | MSI | Unmeth | Wt | Mut | Wt | Wt | Wt |
| 097 | M | 93 | III | Proximal | MSS | Meth | Wt | Wt | Wt | Mut | Wt |
| 002 | M | 75 | I | Rectum | MSS | Unmeth | Wt | Wt | Mut | Wt | Wt |
| 003 | F | 79 | II | Proximal | MSS | Unmeth | Mut | Wt | Wt | Wt | Wt |
| 006 | M | 62 | I | Proximal | MSS | Unmeth | Mut | Wt | Wt | Mut | Wt |
| 008 | F | 39 | I | Distal | MSS | Unmeth | Wt | Wt | Wt | Mut | Wt |
| 009 | F | 62 | I | Distal | MSS | Unmeth | Wt | Wt | Wt | Wt | Mut |
| 013 | F | 59 | II | Proximal | MSS | Unmeth | Mut | Wt | Wt | Wt | Wt |
| 024 | M | 78 | III | Proximal | MSS | Unmeth | Mut | Wt | Wt | Mut | Wt |
| 025 | M | 71 | I | Distal | MSS | Unmeth | Wt | Wt | Wt | Mut | Wt |
| 027 | F | 59 | III | Proximal | MSS | Unmeth | Wt | Mut | Wt | Mut | Wt |
| 029 | F | 66 | II | Distal | MSS | Unmeth | Wt | Wt | Wt | Mut | Wt |
| 036 | M | 35 | III | Rectum | MSS | Unmeth | Wt | Wt | Wt | Mut | Wt |
| 039 | M | 53 | I | Rectum | MSS | Unmeth | Wt | Wt | Wt | Mut | Wt |
| 041 | M | 78 | III | Proximal | MSS | Unmeth | Mut | Wt | Wt | Mut | Wt |
| 042 | M | 84 | II | Proximal | MSS | Unmeth | Wt | Wt | Wt | Mut | Wt |
| 047 | F | 47 | I | Rectum | MSS | Unmeth | Mut | Wt | Wt | Mut | Wt |
| 049 | M | 34 | IV | Rectum | MSS | Unmeth | Wt | Wt | Wt | Mut | Wt |
| 052 | M | 65 | II | Rectum | MSS | Unmeth | Wt | Wt | Wt | Wt | Wt |
| 055 | F | 51 | II | Proximal | MSS | Unmeth | Wt | Wt | Wt | Mut | Wt |
| 057 | M | 67 | I | Rectum | MSS | Unmeth | Wt | Wt | Wt | Wt | Mut |
| 060 | M | 71 | I | Rectum | MSS | Unmeth | Wt | Wt | Wt | Wt | Wt |
| 063 | F | 67 | IV | Distal | MSS | Unmeth | Wt | Wt | Wt | Mut | Wt |
| 065 | F | 91 | II | Proximal | MSS | Unmeth | Mut | Wt | Wt | Mut | Wt |
| 068 | F | 75 | II | Proximal | MSS | Unmeth | Mut | Wt | Wt | Mut | Wt |
| 069 | M | 59 | I | Rectum | MSS | Unmeth | Wt | Wt | Wt | Wt | Wt |
| 070 | M | 58 | III | Distal | MSS | Unmeth | Mut | Wt | Wt | Mut | Wt |
| 071 | M | 85 | I | Rectum | MSS | Unmeth | Mut | Wt | Wt | Mut | Wt |
| 073 | F | 64 | IV | Proximal | MSS | Unmeth | Wt | Wt | Wt | Wt | Wt |
| 074 | M | 73 | I | Proximal | MSS | Unmeth | Wt | Wt | Wt | Mut | Wt |
| 075 | F | 90 | III | Rectum | MSS | Unmeth | Mut | Wt | Wt | Mut | Wt |
| 077 | M | 62 | IV | Distal | MSS | Unmeth | Wt | Wt | Wt | Wt | Wt |
| 082 | M | 78 | IV | Distal | MSS | Unmeth | Wt | Wt | Wt | Wt | Wt |
| 089 | F | 77 | III | Rectum | MSS | Unmeth | Wt | Wt | Wt | Mut | Wt |
| 091 | F | 59 | III | Proximal | MSS | Unmeth | Wt | Wt | Wt | Wt | Wt |
| 092 | M | 85 | III | Proximal | MSS | Unmeth | Mut | Wt | Wt | Wt | Wt |
| 093 | M | 85 | III | Rectum | MSS | Unmeth | Mut | Wt | Wt | Wt | Wt |
| 096 | F | 65 | II | Distal | MSS | Unmeth | Wt | Wt | Wt | Mut | Wt |
| 098 | M | 74 | III | Distal | MSS | Unmeth | Wt | Wt | Wt | Mut | Wt |
| 100 | F | 51 | III | Proximal | MSS | Unmeth | Wt | Mut | Wt | Mut | Wt |
| 107 | M | 58 | I | Distal | MSS | Unmeth | Mut | Wt | Wt | Mut | Wt |
| 108 | F | 87 | I | Proximal | MSS | Unmeth | Wt | Wt | Wt | Mut | Wt |
| 121 | F | 29 | IV | Distal | MSS | Unmeth | Mut | Wt | Wt | Mut | Wt |
| 122 | M | 81 | III | Rectum | MSS | Unmeth | Wt | Wt | Wt | Wt | Wt |
| 123 | F | 81 | II | Rectum | MSS | Unmeth | Wt | Wt | Wt | Mut | Wt |
| 129 | M | 83 | II | Proximal | MSS | Unmeth | Mut | Wt | Mut | Mut | Wt |
| 130 | M | 92 | II | Proximal | MSS | Unmeth | Wt | Wt | Wt | Wt | Wt |
| 134 | M | 58 | III | Rectum | MSS | Unmeth | Mut | Wt | Wt | Mut | Wt |
| 140 | M | 84 | II | Distal | MSS | Unmeth | Mut | Wt | Wt | Wt | Wt |
| 158 | F | 74 | III | Rectum | MSS | Unmeth | Mut | Wt | Wt | Wt | Wt |
| 171 | M | 78 | II | Rectum | MSS | Unmeth | Mut | Wt | Wt | Wt | Wt |
