# Supplementary material for: Phospholipase C Isozymes Are Deregulated in Colorectal Cancer – Insights Gained from Gene Set Enrichment Analysis of the Transcriptome
Source: PLoS One. 2011 Sep 1;6(9):e24419. doi: 10.1371/journal.pone.0024419 (PMC3164721; doi:10.1371/journal.pone.0024419)
Supplement: Table S5 — Primers and probe used for qualitative and quantitative methylation-specific polymerase chain reaction and bisulfite sequencing. For PLCD1 (NM_006225.3) the MSP primers were obtained from Hu et al. (Oncogene, 28, 2009, p.2466-75) whereas the bisulfite sequencing was performed with primers from Hu et al. combined with primers specifically designed for the present study. For PLCE1 (NM_016341), all primers were designed for the present study. Abbreviations: MSP, methylation-specific polymerase chain reaction; M, methylated-specific primers; U, unmethylated-specific primers; qMSP, quantitative methylation-specific polymerase chain reaction; MGB, minor groove binder; BS, bisulfite sequencing. (DOC) [file pone.0024419.s007.doc]

**Table S5**: Primers and probe used for qualitative and quantitative methylation-specific polymerase chain reaction and bisulfite sequencing

|  | Fragment | | 5' primer sequence | 3' primer sequence | Probe | Fragment size | Annealing temperature (°C) |
| --- | --- | --- | --- | --- | --- | --- | --- |
|  | |  |  |  |  |  |  |
| *PLCD1* | | MSP M | AATGATAGGGTTCGCGGTTC | CCCGAACCAACGAACGCG |  | 91 | 58 |
|  | | MSP U | GTAATGATAGGGTTTGTGGTTT | CTAACCCAAACCAACAAACACA |  | 97 | 58 |
|  | |  |  |  |  |  |  |
|  | | qMSP | GTTTCGGGATTATTTTCGGTTTT | ACTTAAATAACGACAACACCGACG | 6FAM-TTGTATCGTATTTCGTTCGGAT-MGB | 101 | - |
|  | |  |  |  |  |  |  |
|  | | BS 1 | GTATTTTTGGGGTTAGAAATT | AAAAACAAAACTAAAAACCC |  | 430 | 52 |
|  | | BS 2 | GYGTTTTTAGAAGGGGG | TCACCRTACAAAATCAAAAA |  | 260 | 50 |
|  | |  |  |  |  |  |  |
| *PLCE1* | | MSP M | GGCGTTAGCGGTTTTTTATTC | CTCCCGCCGAAATATACG |  | 120 | 53 |
|  | | MSP U | GGTGGTGTTAGTGGTTTTTTATTT | CCACTCCCACCAAAATATACA |  | 123 | 53 |
|  | |  |  |  |  |  |  |
|  | | BS | TTTAGGGGTTAGGGTTAGGGGT | ACCCACRTTACCCAACRTTA |  | 327 | 56 |
|  | |  |  |  |  |  |  |
| *ALUC4* | | qMSP | GGTTAGGTATAGTGGTTTATATTTGTAATTTTAGTA | ATTAACTAAACTAATCTTAAACTCCTAACCTCA | 6FAM-CCTACCTTAACCTCCC-MGB | 98 | - |
